# PRO EDI—A Tool to Help Systematic Reviewers Make Equity, Diversity, and Inclusion Assessments

**DOI:** 10.1002/cesm.70083

**Published:** 2026-04-29

**Authors:** Shaun Treweek, Declan Devane, Vivian Welch, Jennifer Petkovic, Peter Tugwell, K. M. Saif‐Ur‐Rahman, Ana Beatriz Pizarro, Agustín Ciapponi, Ioanna Gkertso, Clarinda Cerejo, Hanne Bruhn

**Affiliations:** ^1^ Aberdeen Centre for Evaluation University of Aberdeen Aberdeen UK; ^2^ Department of Women's and Children's Health Uppsala University Uppsala Sweden; ^3^ Evidence Synthesis Ireland & Cochrane Ireland, School of Nursing and Midwifery University of Galway Galway Ireland; ^4^ HRB‐Trials Methodology Research Network University of Galway Galway Ireland; ^5^ Bruyere Research Institute Ottawa Ontario Canada; ^6^ School of Epidemiology and Public Health University of Ottawa Ottawa Ontario Canada; ^7^ Faculty of Medicine University of Ottawa Ottawa Ontario Canada; ^8^ Department of Medicine, Faculty of Medicine University of Ottawa Ottawa Ontario Canada; ^9^ Clinical Epidemiology Program, Ottawa Hospital Research Institute Ottawa Ontario Canada; ^10^ Clinical Research Center, Fundación Valle del Lili Cali Colombia; ^11^ Cochrane Central Executive Team London UK; ^12^ Cochrane Argentina, Instituto de Efectividad Clínica y Sanitaria Buenos Aires Argentina; ^13^ Public Contributor UK; ^14^ Public Contributor, Independent Patient Engagement Consultant Mumbai India; ^15^ Trials Research and Methodologies Unit, HRB Clinical Research Facility University College Cork Cork Ireland; ^16^ HRB‐Trials Methodology Research Network University College Cork Cork Ireland

**Keywords:** equity, diversity and inclusion, evidence applicability, participant characteristics, representation

## Abstract

**Introduction:**

Decisions need evidence, and for healthcare decisions, the evidence decision‐makers often want is a systematic review. However, reviews often lack clarity about who is represented within the evidence they synthesize, which limits understanding of how findings apply to diverse populations. PRO EDI was developed to help systematic review authors extract and report equity‐related participant data to support greater transparency and more informed judgments about applicability.

**Methods:**

PRO EDI was developed iteratively between August 2022 and March 2024 and was conceptualized as a way of making it easier to use PROGRESS‐Plus, a framework to assess equity in reviews. An initial draft was created and then discussed and revised in collaboration with an international advisory group. A relatively mature version of the tool was then presented to a meeting of the Cochrane Health Equity Thematic Group. The modified version that emerged from that meeting was considered v1 of PRO EDI.

**Results:**

PRO EDI has two main components: a participant characteristics table and guidance on how to use the extracted characteristics data within reviews. PRO EDI recommends that six participant characteristics should be extracted for all included studies in a review: age, sex, gender, ethnicity, race and ancestry, socioeconomic status, and location. Other characteristics (e.g., disability) may be important for some reviews. PRO EDI is relevant for all systematic reviews, not just those with an equity focus. The tool has been piloted in several reviews and is publicly available via Trial Forge.

**Conclusion:**

PRO EDI gives systematic review authors a consistent way of deciding which participant characteristics to extract from included studies to support equity‐related judgments in their results and discussion. It also suggests ways in which those judgments can be presented.

## Summary

- Systematic reviews are often not clear about who is represented within a body of evidence.
- Not knowing who is within a body of evidence can leave decision‐makers guessing as to whether the evidence applies to everyone who could benefit, or just some.
- PRO EDI was developed by an international team and gives concrete recommendations for what systematic review authors should extract to describe the participants represented within their review and how to present this information.
- PRO EDI gives guidance on how to use the participant characteristics data to interpret the review findings with regard to applicability of the evidence, as well as equity, diversity and inclusion implications for practice and future research.

## 1 Introduction

It is hard to be confident about a decision made without evidence to support it. This is true for all of life's decisions, whether about washing machines, a defendant's guilt, what to have for dinner, or how to manage a critical illness. What differs is what is considered acceptable evidence.

For health and treatment decisions, the evidence many decision‐makers want is a high‐quality systematic review. Systematic reviews play a central role in informing decisions about what treatment or other healthcare interventions should be delivered, how, and to whom. Organizations such as Cochrane (https://www.cochrane.org/about-us), the Campbell Collaboration (https://www.campbellcollaboration.org/) and their collaborators have created methodological standards for how to do high‐quality reviews [1]. Reporting guidelines like PRISMA [2] describe how the results should be reported. Tools such as ROBUST‐RCT [3], Grading of Recommendations Assessment, Development and Evaluation (GRADE) [4] and Confidence in the Evidence from Reviews of Qualitative Research (CERQual) [5] allow reviewers to make more consistent judgments about evidence certainty. The evidence presented in these reviews is essential for international guidelines, such as those from the World Health Organization (https://www.who.int/), and national guidelines, such as those from the UK's National Institute for Health and Care Excellence (https://www.nice.org.uk/). These collations of evidence‐informed recommendations affect the care of millions of people.

Those people are, obviously, not all the same. In addition to variation in their clinical needs, some will be wealthy, while others will be poor. Some may describe themselves as Indian British, others as Middle Eastern, some as Chinese. There will be men, women and non‐binary genders among them. Importantly, they will not all be in one place and receiving care in the same health system. Characteristics such as sex and ethnicity are often strongly linked to treatment effects and health outcomes [6, 7, 8, 9, 10], which means guidelines need to consider such characteristics.

For that to be possible, the systematic reviews upon which guidelines are based also need to collect information on who appears within the body of evidence and interpret the results accordingly. However, this does not always happen. In their 2022 review of equity reporting in systematic reviews, Welch and colleagues concluded that review authors need to improve reporting of population and setting to support judgments about applicability, both for disadvantaged and other populations not represented in the review [11]. This matters because while the people receiving care may be different from those in the review, if review evidence is presented as though they are the same any inequities in care and outcomes will remain hidden, and in doing so those inequities are likely to persist or widen.

For example, clinical variation like disease severity may have been considered when presenting evidence on the effects of a treatment, but not whether those having more severe disease are, say, predominantly women or from a small number of ethnic groups. Or whether people who share some characteristics (e.g., older black women) have poorer outcomes than people who do not share all or some of these characteristics (e.g., younger white women), sometimes called intersectionality. For example, a (hypothetical) systematic review on the effectiveness of a new diabetes medication may conclude that the drug is highly effective but fail to highlight that:

1. Most studies were conducted in high‐income countries.
2. Participants were predominantly middle‐aged and older adults.
3. There is limited data on the effect of the drug in diverse ethnic groups.
4. Few studies included patients with common comorbidities like obesity or heart disease.

In other words, the review presents a body of evidence that involves a subset of the type of person who may benefit from the drug.

If a review is not transparent about who is represented within its evidence, a decision‐maker may conclude that all will benefit when in fact the evidence only speaks for a subset of those who *could* benefit. By explicitly recognizing the limits of applicability, inequities in care and outcomes can be identified and then addressed. This is good science.

The main objectives of our work were to:

a. Develop a tool that can be used by *all* systematic review authors to routinely collect and present equity‐related data in their reviews.
b. Suggest how review authors can use these data to make judgments about the certainty of the evidence for particular populations.
c. Through (a) and (b), support review users to make more informed judgments about the applicability of review findings to their own contexts.

Our primary target audience is therefore systematic review authors, and we focus on systematic reviews in healthcare treatment and delivery. However, we think our work is relevant to others such as trialists, and to systematic review authors working in public health fields such as nutrition, or town planning and housing.

This work was done as part of the Evidence Synthesis Ireland (https://evidencesynthesisireland.ie/) 2021–2025 plan of work, one component of which was to develop a tool to help systematic review authors build equity, diversity and inclusion considerations into their reviews. This work was to be done together with Trial Forge (https://www.trialforge.org/), an initiative to improve the efficiency of trials, which includes how trial results are presented. Importantly, the tool was intended to be relevant for all systematic reviews, not just equity‐focused reviews.

## 2 Methods

PRO EDI was developed iteratively between August 2022 and March 2024. PRO EDI was conceptualized as a way of making it easier to use PROGRESS‐Plus, an existing framework to assess equity in reviews [12]. PROGRESS‐Plus was not the only equity framework available: EQUALSS GUIDES Multiple [13] is another example, and PRISMA‐Equity provides a reporting standard for reviews focusing on equity [14]. But PROGRESS‐Plus is the most frequently used [15], and it therefore became the starting point for PRO EDI.

The PROGRESS acronym stands for:

- Place of residence (rural or urban)
- Race/ethnicity/culture/language
- Occupation (including unemployed)
- Gender/sex
- Religion
- Education
- Socioeconomic status
- Social capital

The “Plus” in PROGRESS‐Plus represents other characteristics such as age, disability, comorbidities and health literacy.

We called our tool PRO EDI because while based on PROGRESS‐Plus, it is different. In the first version of PRO EDI (see File S1) we tried to add PROGRESS‐Plus into the *Characteristics of included participants* table completed for each study included in a review.

To develop that version further, we established an advisory group in February 2023 comprising additional systematic review authors, review authors with experience of working in the Global South, and public contributors. All members of the advisory group are authors of this article. Group members were drawn from our personal networks and in the case of public contributors, through an open call on Cochrane Engage (https://engage.cochrane.org). Through the advisory group we aimed to increase the range of perspectives involved in development and to ensure that PRO EDI was more likely to be relevant to reviews done globally.

These discussions and tests led to many revisions of our initial version of PRO EDI. Key revisions included:

- Removal of items that were not related to participant characteristics. The original version attempted to structure the whole included studies table, largely modeled on the CONSORT for abstracts reporting standard [16]. For example, we made suggestions for how the trial design and objectives should be described. These were removed to keep PRO EDI focused on participant characteristics as we considered these to be the most feasible items to expect all systematic reviewers to extract and consider in their reviews. We accepted that equity, diversity and inclusion are about more than participant characteristics. For example, societal attitudes, power dynamics and institutional structures are important too. But as most reviews currently say nothing about equity, we thought a better description of who was in each included study, and then interpreting the findings accordingly, would be an important and, crucially, feasible step forward.
- Dividing characteristics into “Mandatory” and “Desirable” items. This change was introduced to reduce the workload for systematic review authors using PRO EDI. Through discussion, we judged some characteristics to be relevant for all reviews, while others may be important for some reviews, but not all. This decision was based on our judgment of the likelihood that reviewers would be able and interested in extracting the information routinely.
- Introducing an “Other” item into the table to highlight characteristics that may be important to a particular review but less so to others. Examples could be religion or whether people with impaired cognitive or physical capacity were included in a study.
- The addition of guidance text within the table to explain the characteristic, provide references where appropriate, and highlight potential challenges. This text also made suggestions for how to complete the item.
- Recognition that we would need to provide additional guidance on how to interpret the data being extracted in the PRO EDI characteristics of included participants table.

By July 2023, we had a version of PRO EDI (see File S2) that we could present to around 20 members of the Cochrane Health Equity Thematic Group (https://www.cochrane.org/about-us/our-global-community/thematic-groups/health-equity#team) at a meeting held in St John's, Newfoundland, Canada. This group is international and comprises members from the Global North and South. Its work has been influenced by the 2022 Cochrane Lecture given by one of the authors, JV, on global health [17, 18]. The participants endorsed the importance of PRO EDI but suggested that some items of PROGRESS‐Plus (and therefore PRO EDI) were more universally important than others. This supported our existing division of items into Mandatory, Desirable and Other, and introduced the idea of a core information set—the mandatory items—for extracting equity‐related participant characteristic data in reviews. Like all core information sets, these items are a minimum: review teams are encouraged to extract additional participant characteristics if they judge them important for their review.

Further feedback (e.g., from members of the UK's National Institute of Health and Care Research Evidence Synthesis Centre Equity, Diversity and Inclusion Working Group, participants in a workshop held at the 2023 Cochrane Colloquium, the authors of Reference [18], as well as from our advisory group) led to further revisions, key among which were:

- Renaming our Desirable category as “Depends on review.”
- Explicitly moving some of the PROGRESS‐Plus “Plus” items out of Other and into the main body of the table as “Depends on review” (e.g., sexual identity) to raise awareness of these characteristics while not making them mandatory. These items were the characteristics that discussants often, but not always, considered important enough to be mandatory. This lack of consensus is why these characteristics were moved out of Other but not classed as mandatory.
- Further developing our guidance for the characteristics of included participants table by:
- Expanding the text for some characteristics to include additional links to external materials and resources.
- Acknowledging that PRO EDI currently does not explicitly handle intersectionality. In other words, it treats participant characteristics as independent, ignoring the fact that a combination of characteristics can interact to lead to worse (or better) health outcomes for the individual. PRO EDI simply asks systematic review authors to bear intersectionality in mind when interpreting the characteristics of included participants table.
- Adding text that acknowledged that collection of ethnicity data is not allowed in some jurisdictions (e.g., Rwanda), or is only possible under exceptions in others (e.g., France). We also added text clarifying the difference between race, ethnicity and ancestry.
- Added text highlighting the difference between sex and gender. We also suggested how review authors could handle the common situation that the terms sex and gender might be used interchangeably in reviews.
- Developing guidance for interpreting the information extracted in the PRO EDI characteristics of included participants tables for studies included in a review.

In March 2024, we released the v1 22/3/2024 version of PRO EDI characteristics of included participants table on the Trial Forge website (https://www.trialforge.org/trial-diversity/pro-edi/). We encouraged people to try out the tool in their own reviews. We also provided a brief evaluation form should anyone want to give us feedback.

## 3 Results

All the latest PRO EDI materials are available at https://www.trialforge.org/trial-diversity/pro-edi/.

PRO EDI has two components:

1. The PRO EDI characteristics of included participant table, together with guidance on how to complete the table.
2. Guidance on how to use the completed participant characteristics data in a systematic review.

### 3.1 The PRO EDI Participant Characteristics Table

Table 1 shows the short version of the v1 22/3/2024 PRO EDI characteristics of the included participants table. The full version contains guidance, both before (mostly context and limitations) and inside the table itself. Table 2 presents an example of guidance provided in the full table, in this case for sex and gender.

**Table 1:** Short version of the PRO EDI characteristics of included participants table.

Item	Mandatory or depends on review?	Explanation
Age	Mandatory	**How to complete this item:** We suggest ideally mean or median years together with an indication of spread such as range or standard deviation
Sex	Mandatory	**How to complete this item:** We suggest using Male, Female and Intersex. Other descriptions of intersex might be found and if so, they should be reported as reported by study authors. If authors have asked a question about trans history, this information should also be extracted
Gender	Mandatory	**How to complete this item**: We suggest using man and woman together with any other gender identities as reported by the included study. If authors have asked a question about trans history, this information should also be extracted. If no other gender identities than “man” and “woman” are reported, review authors should report this
Sexual identity	Depends on review	**How to complete this item:** We suggest using bisexual, gay, lesbian, heterosexual together with any other sexual identities as reported by the included study
Race, ethnicity, and ancestry	Mandatory	**How to complete this item:** We suggest using the categories reported by study authors
Socio‐economic status (SES)	Mandatory	**How to complete this item:** We suggest using the categories used by study authors
Level of education	Depends on review	**How to complete this item:** We suggest ideally mean or median years together with an indication of spread such as range or standard deviation. Alternatively, categories of grades completed as a number and percentage
Disability	Depends on review	**How to complete this item:** This depends on the review but type of disability and severity would be examples of what could be extracted
Location (country/countries of data collection and site coordination)	Mandatory	**How to complete this item:** We suggest reporting country or countries where study participants were recruited, together with the country or countries of study coordination sites. The additional of rural/urban is helpful if provided
Other factors relevant to the review	Depends on review	**How to complete this item:** Depends on the item

**Table 2:** An example of guidance provided in the full PRO EDI participant characteristics table, in this case for sex and gender.

Item	Mandatory or depends on review?	Explanation
Sex	Mandatory	**How to complete this item:** We suggest using Male, Female and Intersex. Other descriptions of intersex might be found and if so, they should be reported as reported by study authors. If authors have asked a question about trans history, this information should also be extracted. Sex and gender are different. “Sex” is usually a classification as male, female or intersex assigned at birth based on visual anatomy assessment (see https://pubmed.ncbi.nlm.nih.gov/35725304/). Sex is not binary. Sex is often thought of as an exclusively biological characteristic but it is a social construct in that it is based on an expectation of what bodies should look like. Understanding of sex may vary between countries and cultures. Language will change over time (and place) and the best approach is to use the terminology used in included studies but highlight any limitations this may introduce to the review as a whole Sex and gender are often incorrectly used interchangeably, i.e., sometimes “sex” is listed but gender is reported (i.e., woman/man/non‐binary instead of female/male/intersex). We err towards recommending that review teams correct “sex” to “gender” or “gender” to “sex” if required and then state “Corrected to gender (or sex) by the review team.” If the term ‘sex’ isn't explicitly used in the study, but female/male/intersex reported, state “Listed as sex by the review team.” It is of course unclear how a person in the included study would respond to a question headed, say, “Gender” but was presented with options widely considered to represent sex Regardless, review authors need to make a decision about how to handle interchangeable use of sex and gender, and then follow that decision consistently in their review For children (“boys” and girls”) see “Gender” below. **Examples** A set of examples is available at [to follow] **Suggested English search terms to use when searching for this information in a study** [sex/male/female/trans]
Gender	Mandatory	**How to complete this item**: We suggest using man and woman together with any other gender identities as reported by the included study. If authors have asked a question about trans history, this information should also be extracted. If no other gender identities than “man” and “woman” are reported, review authors should report this Sex and gender are different. Gender is a social configuration that gathers the roles, behaviors, activities, feelings, attitudes and attributes that a given society typically associates with being masculine or feminine (see https://pubmed.ncbi.nlm.nih.gov/35725304/). Gender is not binary, nor is understanding of gender the same across the world Language will change over time (and place) and the best approach is to use the terminology used in included studies but highlight any limitations this may introduce to the review as a whole. Try to avoid using “non‐binary” as a catch all category if possible as some experience this practice as harmful Sex and gender are often incorrectly used interchangeably, i.e., sometimes “sex” is listed but gender is reported (i.e., woman/man/non‐binary instead of female/male/intersex). We err towards recommending that review teams correct “sex” to “gender” or “gender” to “sex” if needed and then stating “Corrected to gender (or sex) by the review team.” If the term “sex” isn't explicitly used in the study, but female/male/intersex reported, state “Listed as sex by the review team.” If only binary genders are reported, assume trans history was not collected, add: “It is unclear whether and how people with a trans history would answer a binary gender question.” Also see “Sex” above When “boys” and “girls” are used for children, it should be listed as “gender” and include other gender identities. It should be acknowledged if the reviewers have corrected this from what was reported in the study **Examples** A set of examples is available at [to follow] **Suggested English search terms to use when searching for this information in a study** [gender/man/woman/trans]

### 3.2 Guidance on Places to Use Participant Characteristics Data in a Systematic Review

There are at least three places in a review where the information from the PRO EDI participant characteristics table should be considered:

1. An overall summary table of who is included in the review. This shows (a) who would be expected to be represented in the review based on the disease, prevalence, severity, exposure, etc. and (b) those represented in the review.
2. The synthesis of results for each comparison.
3. The discussion.

For some of what follows we will use a systematic review on falls prevention [19] as the basis of our examples because we used this review as a test‐case when developing PRO EDI. It is worth emphasizing that we were not involved in this review, that we are not experts in falls prevention, and that the authors of the review have not been involved in the presentations in this paper. We have not chosen this review because we are critical of it, far from it. We chose it because it was a good review to work with when developing PRO EDI. Any errors in the presentations here are ours, not those of the review authors.

**Figure 1:**
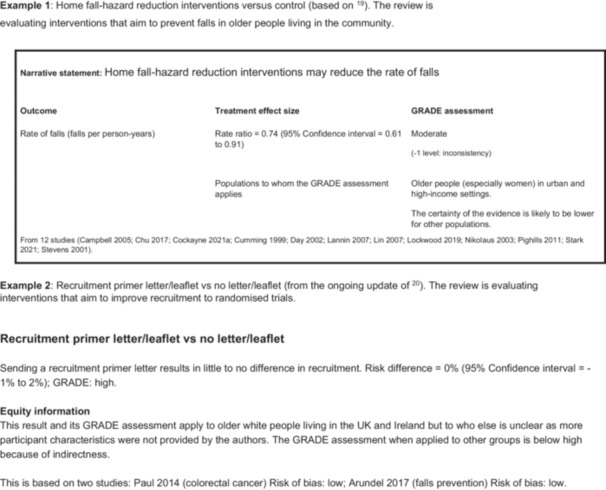
Examples of how equity‐related assessment information from PRO EDI could be presented.

**Figure 2:**
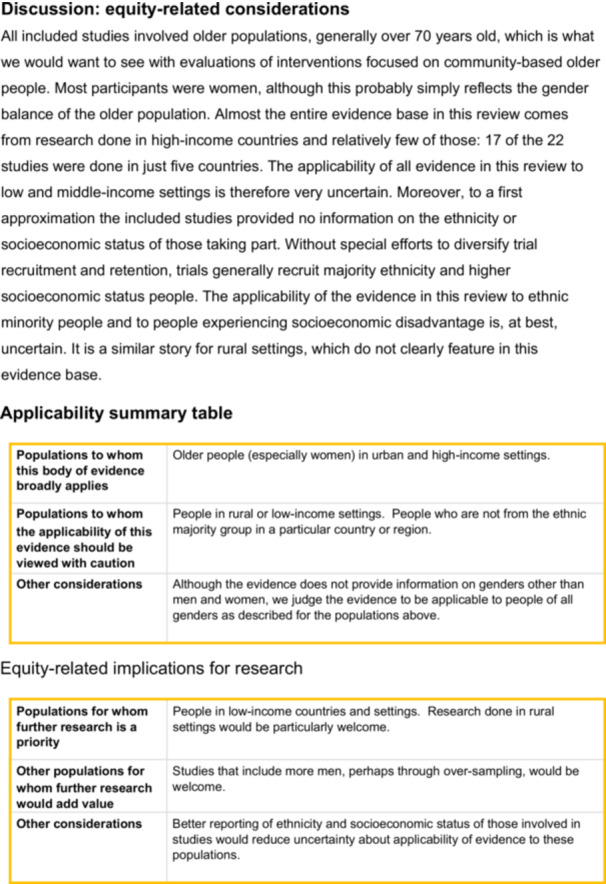
Example of a narrative assessment of the equity and applicability implications of the participants included in a body of evidence.

1. An overall summary table of who is included in the review
  A summary table gives users of the review an overview of the extent to which the body of evidence presented in the review includes the people expected given the health condition. To complete the *people we would expect to see included* column the review team could examine the relevant literature on, say, prevalence or incidence, or they could use their existing knowledge of the field, or a combination. This will inevitably include some subjective judgment. The intention is not to give review teams a substantial additional piece of work, but for them to consider who they would expect to see in the included studies, and to do the best job they can with the time and resources at their disposal.
  Important differences between who was expected and who is actually represented in the review should be raised in the Discussion (see below). Users of the review can then build this into their judgments about applicability.
  Table 3 shows the example summary table from our PRO EDI guidance, based on the falls review [19]. The PRO EDI characteristics of included participants tables for the included studies behind Table 3 are available in File S3.
  An important thing to note about Table 3 is that the text in the *People who took part* column is not a blow‐by‐blow account of each included study but an overall assessment by the systematic review authors. The example also highlights two other things. Firstly, review authors may choose to not extract some of the participant characteristics that we class as “depends on the review” because they do not think the characteristic will have a bearing on the outcomes reported in the review. Sexual identity falls into this category in Table 3, which records “No extraction.” Secondly, review authors may aim to extract information but find little to extract. Ethnicity falls into this category in Table 3. There is little review authors can do about this, but it is important to flag the limitation so that users of the review know about it.
2. Synthesis of results for each comparison
  We think it is important to attach an equity‐related assessment to the results presented for each comparison presented in a review. This means that there is an assessment reported in close proximity to the result and not only at the end of the Discussion section.
  PRO EDI is not mature enough for us to be prescriptive about how this assessment is presented but Figure 1 present two examples. The first is based on the falls review [19], the second shows how we are presenting equity‐related assessments in the ongoing update of the 2018 Cochrane recruitment review [20].
  These are examples, not recommendations. The central message of Figure 1 is that along with the presentation of the review finding, there is also an explicit description as to whom the review authors think this finding applies. Both examples link this to the GRADE [4] assessment of the certainty of the evidence; in Figure 1 we show how the rating of certainty can be indicated in a table, based on PRO EDI assessments and other information.
3. In the discussion
  The discussion section of a review must cover many things, but one of them should be an equity‐related assessment of the evidence that has been reviewed. This is most likely to be a narrative assessment of the applicability implications of the participants included in a body of evidence, ideally with reference to who would be expected in the review given disease prevalence.
  Figure 2 shows the example from our PRO EDI guidance for the falls study [19]. After a narrative statement informed by the summary table shown in Table 3, the example provides two tabulated summaries: one an applicability summary table and the other an equity‐related implications for research table. The intention is that together these provide review users with a sense of to whom the evidence applies, and to whom it does not, along with how future research could address equity‐related limitations.

**Table 3:** Example of an overall summary table of the participant characteristics we would expect to see in the evidence and the actual participant characteristics extracted from the included studies.

PRO EDI participant characteristics		
Characteristic	The people we would expect to see included	The people who took part
**Age**	A third of people over 65 fall each year, with the risk of fall‐related injuries increasing with age. We would expect participants who are older; average age expected to be over 65	All included studies involved people who were older, with mean ages generally over 70 and often 80
**Sex**	All sexes	Where sex was reported, the majority of participants were female, always over 50% and frequently over 70%
**Gender**	Affects all genders. Women have a high risk of indoor falls, men more likely to fall in presence of outdoor environmental hazards	Where gender was reported, the majority of participants were women, almost always over 50% and frequently over 70%
**Sexual identify**	Other characteristics were prioritized for this review	No extraction
**Ethnicity**	Links have been made to ethnicity and falls, largely mediated through socioeconomic status and poorer housing. We would expect participants with a mix of ethnicity reflective of where the study was done	Inadequate information: the included studies say little or nothing about the ethnicity of participants
**Socioeconomic status**	Links have been made between lower socioeconomic status and falls linked to poorer housing. People experiencing socioeconomic disadvantage should be included	A small number of studies did involve people experiencing socioeconomic disadvantage. Most included studies say little or nothing about the socioeconomic status of participants
**Level of education**	Other characteristics were prioritized for this review	No extraction
**Disability**	Other characteristics were prioritized for this review	No extraction
**Location**	Falls are a problem seen across the world. We would expect to find studies from around the world in a mix of urban and rural environments	Almost all studies were done in high income countries, with Australia (7), USA (4), New Zealand (2), France (2) and the UK (2) accounting for 17 of the 22 studies. The included studies therefore say little or nothing about falls interventions in low and middle‐income countries. Whether studies involved urban and rural environments was rarely reported. However, when a study did provide information about local locations, these were generally urban
**Other**	No other characteristics were considered	No extraction

*Note:* This example is taken from the PRO EDI guidance, which uses a review on preventing falls in older people living in the community [19].

## 4 Use of PRO EDI

The team updating the 2018 Cochrane review of interventions to improve trial recruitment [20], led by the University of York, UK, and involving ST and HB, agreed to use PRO EDI in the update. PRO EDI was used in a scoping review [21] and a systematic review of the effectiveness of SARS‐CoV‐2 testing strategies [22] and will be used in a review of automated and manual measurements of mammographic breast density in individuals undergoing breast cancer screening [23]. PRO EDI is also being evaluated in a Study Within A Review (SWAR) as part of a study looking at clinical practice guidelines for end‐stage kidney failure [24] and as part of SWAR embedded in a systematic review of published heart failure research [25]. Use of PRO EDI when reporting trial protocols and trial reports has also been mandated by the journal *Trials* [26]. Finally, PRO EDI is being fully incorporated into broader guidance on considering and reporting methods for investigating health equity issues in systematic reviews and other evidence syntheses that is currently being developed by the Evidence for Policy and Practice (EPPI) Centre and others [27].

## 5 Discussion

PRO EDI gives systematic review authors a consistent way of deciding which participant characteristics to extract from included studies to support equity‐related judgments in their results and discussion. It also suggests ways in which those judgments can be presented. We think it is relevant for all systematic reviews.

We believe PRO EDI has some important strengths. It uses one of the world's most‐used equity‐related evidence synthesis tools, PROGRESS‐Plus [12], and provides specific guidance on how to operationalize it. In summary, operationalisation boils down to extract the PRO EDI characteristics and report what you find.

PROGRESS‐Plus (and therefore PRO EDI) is not perfect. Two 2023 critiques of PROGRESS‐Plus [28, 29] discuss its limitations and reach a similar conclusion: that a more detailed conceptual framework is needed that better reflects the complexity of inequality and inequity. In 2024, Hollands and colleagues [15] concluded that PROGRESS‐Plus was sufficiently broad to examine dimensions of equity, but they were less certain as to whether it could guide critical thinking in more complex pathways, including between dimensions of equity (a point also made by McCann and colleagues [28]).

Inequality and inequity are likely to be beyond the reach of any single tool or framework. Our more limited goal was to produce a tool that could help systematic review authors do a better job of considering equity than they do at present. We explicitly did not want to develop a tool that would only be used by those already enthusiastic about equity considerations: we wanted a tool that could be routinely used by reviewers working on *any* systematic review in healthcare treatment and delivery. The current equity‐consideration baseline is low. Try selecting a systematic review at random and searching for ethnicity or socioeconomic status, and you'll almost certainly find nothing. Improving that baseline needs acceptable simplicity; complexity can come later when the baseline has shifted.

PRO EDI has been developed iteratively and with a broad range of relevant parties, including public contributors. As with any development, the perspectives sought could have been broader still, but we do think we did a reasonable job given time and financial constraints. PRO EDI is concrete: the characteristics of the included participants table have a clear structure and take some guesswork out of decisions about what to extract. Consistently extracting PRO EDI characteristics means reporting becomes more consistent. Our guidance highlights some of the practical, cultural and legal challenges that trialists face when collecting participant characteristics. Trialists may have been unable or prevented from collecting the participant characteristics we recommend as mandatory. If that is the case, review authors will in turn be unable to extract these data. How characteristics are collected may vary across jurisdictions, giving reviewers the unenviable task of trying to find order in multiple ways of describing the same characteristic.

PRO EDI accepts these realities and is designed to work with them, rather than pretend that the world is not complicated. We see this as a strength. Similarly, the PRO EDI interpretation guidance helps to take some of the guesswork out of what to say (and how) about equity‐related implications for the applicability of the review findings. We think PRO EDI can be integrated into existing systematic review processes to support tasks that reviewers are already trying to do. It provides structure, help and guidance. There is no denying that using PRO EDI represents work. However, systematic review authors always need to describe the people in their reviews. In our experience, data extraction only slows down when authors of included trials provide very good information about who was in their trial, and that information seems worth extracting. Finally, although we have focused on systematic reviews of randomized evaluations of health treatments, we think it has relevance to any review involving studies about people.

This is, however, v1 of PRO EDI, which implies that while we think it is useful, we know there are things it could be doing better. In short, there are limitations. For example, PRO EDI currently asks reviewers to consider intersectionality but does not give concrete help. We have six mandatory characteristics, but we also say that for some reviews other characteristics may be important; we do not say how these characteristics should be selected. We give no guidance on how equity‐related concerns should influence or modify the review's statistical approach. We say nothing about who should be in the review team. Our interpretation guidance says that the content of the characteristics of included participants table may lead reviewers to adjust their GRADE assessments for some participants and contexts, but we don't specifically say how. On this latter point we think it is better to work with the GRADE Equity Working Group [4, 30] to develop formal guidance rather than for us to attempt it here. We are in touch with the GRADE Working Group, and guidance will be developed. These limitations are important but tackling them will take time. We prioritized releasing v1 of PRO EDI because we think the current version is useful as it is, and fully addressing the limitations we mention represents many more years of work. By releasing PRO EDI now, others can join us in addressing the challenges we raise while in the meantime review authors can use PRO EDI to better describe and discuss who is represented in their reviews.

PRO EDI is not therefore the be‐all and end‐all of equity, diversity and inclusion in systematic reviews. Like Rome, effective and consistent handling of equity, diversity and inclusion is not built in a day. Much remains to be done, and others are working in this area, notably the Campbell and Cochrane Health Equity Thematic Group (https://www.cochrane.org/about-us/our-global-community/thematic-groups/health-equity#team) and the EPPI Centre [27].

However, we think PRO EDI does two important things and does them rather well. First, it tells reviewers how to describe who is included in their evidence and, second, how to draw review users’ attention to any applicability limitations that may follow. If systematic review authors use PRO EDI, it ought to be harder for review users to conclude that a body of evidence applies to everyone when in fact it has only been evaluated for some. Evidence may apply to populations not seen within review, but this is a judgment that should be explicit, not implicit. We think PRO EDI helps to ensure this.

There is another use for the PRO EDI characteristics of included participants table: the minimum information set of participant characteristics to be collected in a randomized trial. As a systematic review author using PRO EDI will quickly discover, “No information provided by authors” will become a common entry in their data extraction forms. The equity‐related data collected and reported in trials must improve [15, 31, 32, 33]. If a trial team is unsure which participant characteristics to collect, PRO EDI provides an answer: collect the mandatory items in the PRO EDI characteristics of included participants table. This will reduce variability in data collection and improve reporting. It is unlikely that PRO EDI is only relevant for trials too.

That won't be enough for everyone. But if more trialists did this, and funders and journals started to expect it, everyone who works with trials and systematic reviews would know much more about who is included in our evidence than we do today. For the future, in addition to collaboration with the GRADE Working Group, we intend to work with other groups to see to what extent PRO EDI can support consistent consideration of equity, diversity and inclusion in systematic reviews. For example, Cochrane requires authors to provide information on how equity will be addressed in reviews. Some of us have developed two tutorials [34, 35] to support authors meet this requirement and the second of these recommends use of PRO EDI [35]. More concrete guidance on topics such as intersectionality will come, as will training resources. There will be a v2 of PRO EDI.

To conclude, we encourage systematic review authors and trialists to use PRO EDI, and feedback on it is welcome using the evaluation form at https://www.trialforge.org/trial-diversity/pro-edi/, or by simply emailing us at info@trialforge.org.

## Author Contributions

**Shaun Treweek:** conceptualization, investigation, funding acquisition, methodology, writing – original draft, validation, writing – review and editing, project administration, data curation, resources, formal analysis. **Declan Devane:** conceptualization, methodology, investigation, funding acquisition, writing – review and editing, validation, resources. **Vivian Welch:** conceptualization, writing – review and editing, methodology, investigation, validation. **Jennifer Petkovic:** conceptualization, investigation, methodology, validation, writing – review and editing. **Peter Tugwell:** conceptualization, investigation, methodology, validation, writing – review and editing. **K. M. Saif‐Ur‐Rahman:** investigation, methodology, validation, writing – review and editing. **Ana Beatriz Pizarro:** investigation, methodology, validation, writing – review and editing. **Agustín Ciapponi:** investigation, methodology, validation, writing – review and editing. **Ioanna Gkertso:** investigation, methodology, validation, writing – review and editing. **Clarinda Cerejo:** investigation, methodology, validation, writing – review and editing. **Hanne Bruhn:** conceptualization, investigation, methodology, validation, writing – review and editing, project administration, data curation, formal analysis.

## Ethics Statement

Ethical approval was not required for this work. Members of the public who took part did so as patient and public partners helping to drive and shape the work, not as research participants.

## Conflicts of Interest

Shaun Treweek has acted as a paid consultant on trial diversity to the National Institute for Health and Care Research (NIHR) Oxford Health Biomedical Research Centre and the NIHR Cambridge Biomedical Research Centre. All other authors declare no conflicting interests.

## Patient and Public Involvement

We have two public contributor co‐authors, Ioanna Gkertso and Clarinda Cerejo, who have been actively engaged throughout the development of PRO EDI. Ioanna Gkertso and Clarinda Cerejo have also directly participated in dissemination events about PRO EDI.

## Supporting information

Supporting File 1

Supporting File 2

Supporting File 3

## Data Availability

The data that supports the findings of this study are available in the supplementary material of this article.
