# Supplementary material for: PRO EDI—A Tool to Help Systematic Reviewers Make Equity, Diversity, and Inclusion Assessments
Source: Cochrane Evid Synth Methods. 2026 Apr 29;4(3):e70083. doi: 10.1002/cesm.70083 (PMC13131102; doi:10.1002/cesm.70083)
Supplement: Supplementary file 1 — Supporting File 1 [file CESM-4-e70083-s001.docx]

**Characteristics of included studies table template**

**6/1/2023**

| Heading | Explanation | | Why this heading? |
| --- | --- | --- | --- |
| **Title** | Identification of the study as randomized | | CONSORT for abstracts  Provided elsewhere in review |
| **Authors** | Contact details for the corresponding author | | CONSORT for abstracts  Provided elsewhere in review |
| **Trial status** | Is the trial ongoing, closed to recruitment, or closed to follow-up (i.e. complete) | | CONSORT for abstracts |
| **Trial design** | Description of the trial design (e.g. parallel, cluster, non-inferiority) | | CONSORT for abstracts (<https://www.equator-network.org/reporting-guidelines/consort-abstracts/>) |
| **Objective** | Specific objective or hypothesis of the trial | | CONSORT for abstracts |
| **Methods** | | |  |
| ***Participants targeted for trial*** | Eligibility criteria for participants | | Modified CONSORT for abstracts (CONSORT asks for setting but we ask for this in our PROGRESS-Plus equity bundle) |
| ***Interventions and comparators*** | Interventions intended for each group | | CONSORT for abstracts |
| ***Outcomes*** | Summary of the primary and secondary outcomes | | Modified CONSORT for abstracts (CONSORT just asks for primary) |
| ***Randomisation*** | How participants were allocated to interventions | |  |
| ***Blinding (masking)*** | Whether or not participants, care givers, and those assessing the outcomes were blinded to group assignment. Describe who (e.g. participants, healthcare providers, outcome assessors, data analysts) was blinded rather than saying ‘single’, ‘double’ etc. It may not alway be possible to blind some people involved in the trial but if there is any form of blinding, it should be reported. | | CONSORT for abstracts |
| **Results** | | |  |
| ***Participants in trial*** | Describe the characteristics of the participants involved in the trial under the subheadings listed below. | | PROGRESS-Plus (<https://methods.cochrane.org/equity/projects/evidence-equity/progress-plus>) |
|  | **Subheading** | **Explanation** |  |
|  | *Age* | Years. Mean or median together with an indication of spread such as range. | Widely considered important and routinely reported. To raise awareness of the exclusion of age extremes (i.e. the very young and the very old). |
|  | *Sex* | Male; female; intersex | PROGRESS-Plus |
|  | *Gender* | Man; woman; non-binary | PROGRESS-Plus |
|  | *Ethnicity* | The breakdown should be as detailed as trial reports allow. Reviewers should avoid aggregating categories (e.g. ‘Non-White’) | PROGRESS-Plus |
|  | *Socioeconomic status* | The breakdown should be as detailed as trial reports allow. May be categories for some countries, income in others, and proxy measures in others. Reviewers should aim to provide as much detail as they can. | PROGRESS-Plus |
|  | *Level of education* | As for socioeconomic status | PROGRESS-Plus |
|  | *Location* | Country or countries | PROGRESS-Plus |
|  | *Cognitive impairement/capacity to consent* | Describe whether individuals with cognitive impairment/impaired capacity to consent were included in the trial. | To raise awareness around trial consent/eligibility processes that are often used for convenience but which systematically exclude large numbers of people from research participation and, as a consequence, their perspective does not shape health care delivery. |
|  | *Other factors relevant to the review* | Describe other factors that are important for the review. | All reviews are different. There may be factors that need to be reported for some reviews but not others. |
| ***Numbers randomised*** | Number of participants randomised to each group | | CONSORT for abstracts |
| ***Numbers analysed*** | Number of participants analysed in each group | | CONSORT for abstracts |
| ***Outcome*** | The primary outcome result for each group giving the estimated effect size and its precision. Similar information for secondary outcomes that are important for the review as judged by the review authors. | | Modified CONSORT for abstracts (CONSORT just asks for primary)  Provided elsewhere in review |
| ***Harms*** | Important adverse events or side effects | | CONSORT for abstracts  Provided elsewhere in review |
| **Conclusions** | General interpretation of the results | | CONSORT for abstracts  Trial author interpretation not relevant for review |
| **Trial registration** | Registration number and name of trial register | | CONSORT for abstracts |
| **Funding** | Source of funding | | CONSORT for abstracts |
| **Other** | Any other relevant information. | | All reviews are different. There may be information that needs to be reported for some reviews but not others. |
