# Supplementary material for: PRO EDI—A Tool to Help Systematic Reviewers Make Equity, Diversity, and Inclusion Assessments
Source: Cochrane Evid Synth Methods. 2026 Apr 29;4(3):e70083. doi: 10.1002/cesm.70083 (PMC13131102; doi:10.1002/cesm.70083)
Supplement: Supplementary file 2 — Supporting File 2 [file CESM-4-e70083-s002.docx]

**Characteristics of included studies table template**

1. This table is designed for included studies that are randomised trials. We recognise that the table will need to be modified to support other types of included studies.
2. We recognise that many, perhaps most included trials may not report all the items requested in this table especially the items that describe the trial participants. Our intention is that reviewers and review users will interpret trial evidence in light of these items and where information is lacking (e.g. around the characteristics of the people in the trial) that this uncertainty is made clear. We also hope that this may influence how trials are designed and reported in the future.
3. .
4. There is detailed guidance for how to complete this table if you want it here: [insert title of document and link/appendix]

| Heading | Explanation | | Why this heading? |
| --- | --- | --- | --- |
| **Trial status** | Is the trial ongoing, closed to recruitment, or closed to follow-up (i.e. complete) | | CONSORT for abstracts |
| **Trial design** | Description of the trial design (e.g. parallel, cluster, non-inferiority)  Number of study arms in the study and in this review | | CONSORT for abstracts (<https://www.equator-network.org/reporting-guidelines/consort-abstracts/>)  Cochrane  MECIR Manual  Description of studies (R56-R72) |
| **Methods** | | |  |
| ***Participants targeted for trial and potential participants purposefully excluded*** | Inclusion Criteria:  These are the specific characteristics or conditions that participants must have to be included in the study. | | Modified CONSORT for abstracts (CONSORT asks for setting but we ask for this in our PROGRESS-Plus equity bundle) |
|  | Exclusion Criteria:  These are the specific characteristics or conditions that disqualify potential participants from being included in the study. They may include: age, language, cognitive status (e.g. dementia), comorbidities etc. | |  |
| ***Interventions*** | Interventions intended for each group  How to complete this item:  Intervention 1: describe what (materials) and the procedure (how) and how often the intervention is administered  Intervention 2: describe what (materials) and the procedure (how) and how often the intervention is administered  etc | | CONSORT for abstracts  TIDieR |
| ***Comparators*** | Comparators used for each control group  How to complete this item:  Comparator 1: describe what (materials) and the procedure (how) and how often the comparator is administered  Comparator 2: : describe what (materials) and the procedure (how) and how often the comparator is administered | | CONSORT for abstracts  TIDieR |
| ***Outcomes*** | Summary of the primary and secondary outcomes | | Modified CONSORT for abstracts (CONSORT just asks for primary) |
| ***Randomisation*** | How participants were allocated to interventions  [not amended this as in Risk of Bias table close to Characteristics of included studies table] | | CONSORT for abstracts  Provided elsewhere in review – captured in risk of bias assessment |
| ***Blinding (masking)*** | Whether or not participants, care givers, and those assessing the outcomes were blinded to group assignment. Describe who (e.g. participants, healthcare providers, outcome assessors, data analysts) was blinded rather than saying ‘single’, ‘double’ etc. It may not alway be possible to blind some people involved in the trial but if there is any form of blinding, it should be reported.  [not amended this as in Risk of Bias table close to Characteristics of included studies table] | | CONSORT for abstracts  Provided elsewhere in review – captured in risk of bias assessment |
| **Results** | | |  |
| ***Participants in trial*** | Describe the characteristics of the participants involved in the trial under the subheadings listed below. | | PROGRESS-Plus (<https://methods.cochrane.org/equity/projects/evidence-equity/progress-plus>) |
|  | **Subheading** | **Explanation** |  |
| Mandatory | *Age* | Years. Mean or median together with an indication of spread such as range or standard deviation. | Widely considered important and routinely reported. To raise awareness of the exclusion of age extremes (i.e. the very young and the very old). |
| Mandatory | *Sex* | Male; female; intersex. It is important to note that sex and gender are different. ‘Sex’ is usually a classification as male, female or intersex assigned at birth based on visual anatomy assessment (see <https://pubmed.ncbi.nlm.nih.gov/35725304/>). Sex is not binary. Sex is often thought of as an exclusively biological characteristic but it is a social construct in that it is based on an expectation of what bodies should look like. Understanding of sex may vary from country to country.  Language will change over time (and place) and the best approach is to use the terminology used in included studies but highlight any limitations this may introduce to the review as a whole.  How to complete this item:  Sometimes sex is listed but gender is reported (i.e. woman/man/non-binary). When completing this table, correct it to gender and state ‘Corrected to gender by the review team’. If sex isn’t explicitly used in the study, but female/male/intersex reported state ‘listed as sex by the review team’. | PROGRESS-Plus |
| Mandatory | *Gender* | Man; woman; non-binary. It is important to note that gender and sex are different. Gender is a social configuration that gathers the roles, behaviours, activities, feelings, attitudes and attributes that a given society typically associates with being masculine or feminine (see <https://pubmed.ncbi.nlm.nih.gov/35725304/>). Gender is not binary, nor is understanding of gender the same across the world.  Language will change over time (and place) and the best approach is to use the terminology used in included studies but highlight any limitations this may introduce to the review as a whole.  How to complete this item:  Sometimes gender is listed but sex is reported (i.e. female/male/intersex). When completing this table, correct it to sex and state ‘Corrected to sex by the review team’. If gender isn’t explicitly used in the study, but woman/man/non-binary reported state ‘Listed as gender by the review team’.  When ‘boys’ and ‘girls’ are used for children, it should be listed as gender and acknowledged if the reviewers have corrected this. | PROGRESS-Plus |
| Mandatory | *Race, ethnicity and ancestry* | The breakdown should be as detailed as trial reports allow. Reviewers should avoid aggregating categories (e.g. ‘Non-White’) but reported what is listed in studies.  What is meant by ethnicity varies across jurisdictions. Reviewers will need to be clear about how they chose to define ethnicity. | PROGRESS-Plus |
| Madatory | *Socioeconomic status* | The breakdown should be as detailed as trial reports allow. Categories may be used in some countries (e.g. Scotland uses a location-based measure called the Scottish Index of Multiple Deprivation), participant income bands might be used in others (e.g. participant income above or below certain amounts), or proxy measures (e.g. newspapers read by participants has been used to assess socioeconomic status in some studies). Reviewers should aim to provide as much detail as they can.  [not sure how to address [name’s] important point] | PROGRESS-Plus |
| Highly desirable | *Level of education* | As for socioeconomic status | PROGRESS-Plus |
| Mandatory (country/countries)) | *Location* | Country or countries and Rural/Urban  Reviewers should not categorise locations into rural/urban themselves but only use these or equivalent categories if reported in the study. | PROGRESS-Plus |
| Highly desirable | *Other factors relevant to the review* | Describe other factors that are important for the review. For example, whether individuals with cognitive impairment/impaired capacity to consent, communication challenges or other types of disability were included, or whether people of different religions or occupations where access to healthcare or outcomes may differ because of religion or occupation were included.  [maybe this doesn’t make so much sense as we are asking for the exclusion criteria? Maybe better to ask for – Describe other factors that are important for the review. This could include whether the study took steps to be inclusive of certain age groups/ethnic groups/people with cognitive impairment/impaired capacity to consent, communication challenges or other types of disability or whether people of different religions or occupations where access to healthcare or outcomes may differ because of religion or occupation were considered.] | PROGRESS-Plus and all reviews are different. There may be factors that need to be reported for some reviews but not others. |
| ***Numbers randomised*** | Number of participants randomised to each group and, in addition, the proportion of those screened for eligibility that the total number randomised represents. | | CONSORT for abstracts; to present information on trial selectivity. |
| **Trial registration** | Registration number and name of trial register | | CONSORT for abstracts |
| **Funding** | Source of funding | | CONSORT for abstracts |
| **Other** | Any other relevant information. | | All reviews are different. There may be information that needs to be reported for some reviews but not others. |
